# Pelvic floor muscle contraction automatic evaluation algorithm for pelvic floor muscle training biofeedback using self-performed ultrasound

**DOI:** 10.1186/s12905-024-03041-y

**Published:** 2024-04-04

**Authors:** Miyako Muta, Toshiaki Takahashi, Nao Tamai, Motofumi Suzuki, Atsuo Kawamoto, Hiromi Sanada, Gojiro Nakagami

**Affiliations:** 1 Department of Gerontological Nursing / Wound Care Management, Graduate School of Medicine, The University of Tokyo, 7-3-1, Hongo, Bunkyo-ku, Tokyo Japan; 2 Department of Nursing, Yokohama City University, 3-9, Fukuura, Kanazawa-ku, Yokohama-shi, Kanagawa Japan; 3 Department of Urology, Tokyo Metropolitan Bokutoh Hospital, 4-23-15, Kotobashi, Sumida-ku Tokyo, Japan; 4 Division of Ultrasound, Department of Diagnostic Imaging, Tokyo Medical University Hospital, 6-7-1, Nishishinjuku, Shinjuku-ku Tokyo, Japan; 5 Ishikawa Prefectural Nursing University, 1-1, Gakuendai, Kahoku-shi, Ishikawa Japan; 6 Global Nursing Research Center, Graduate School of Medicine, The University of Tokyo, 7-3-1, Hongo, Bunkyo-ku Tokyo, Japan; 7 Department of Urology, The Kikkoman General Hospital, 100, Miyazaki, Noda-shi, Chiba Japan

## Abstract

**Introduction:**

Non-invasive biofeedback of pelvic floor muscle training (PFMT) is required for continuous training in home care. Therefore, we considered self-performed ultrasound (US) in adult women with a handheld US device applied to the bladder. However, US images are difficult to read and require assistance when using US at home. In this study, we aimed to develop an algorithm for the automatic evaluation of pelvic floor muscle (PFM) contraction using self-performed bladder US videos to verify whether it is possible to automatically determine PFM contraction from US videos.

**Methods:**

Women aged ≥ 20 years were recruited from the outpatient Urology and Gynecology departments of a general hospital or through snowball sampling. The researcher supported the participants in their self-performed bladder US and videos were obtained several times during PFMT. The US videos obtained were used to develop an automatic evaluation algorithm. Supervised machine learning was then performed using expert PFM contraction classifications as ground truth data. Time-series features were generated from the x- and y-coordinate values of the bladder area including the bladder base. The final model was evaluated for accuracy, area under the curve (AUC), recall, precision, and F1. The contribution of each feature variable to the classification ability of the model was estimated.

**Results:**

The 1144 videos obtained from 56 participants were analyzed. We split the data into training and test sets with 7894 time series features. A light gradient boosting machine model (Light GBM) was selected, and the final model resulted in an accuracy of 0.73, AUC = 0.91, recall = 0.66, precision = 0.73, and F1 = 0.73. Movement of the y-coordinate of the bladder base was shown as the most important.

**Conclusion:**

This study showed that automated classification of PFM contraction from self-performed US videos is possible with high accuracy.

## Introduction

The International Continence Society defines urinary incontinence (UI) [1] as “the complaint of any involuntary leakage of urine.” UI is common in women, because pregnancy and childbirth are risk factors [2]. The estimates of UI prevalence in women vary between 25% and 45% in most studies [3]. The physical and psychological effects of UI lower a patient’s quality of life [4]. Therefore, adult women should prevent and improve their UI symptoms. Pelvic floor muscle training (PFMT) is recommended to prevent and improve stress, urgency, and mixed UI [5].

Because pelvic floor muscles (PFM) cannot visualized and struggle to contract properly [6], PFMT in combination with biofeedback is effective [7, 8]. Biofeedback involves visual or auditory feedback to gain control over involuntary bodily functions. Perineometry and electromyography [9, 10] are common biofeedback methods used in PFMT. They are invasive because they require the insertion and application of instruments into the vagina and anus. Currently, biofeedback methods are mainly performed in hospitals because they require specialized equipment and techniques. As PFMT needs to be continued mainly at home for continuous training [11], an easy and non-invasive biofeedback method available at home by themselves that can enable the patient to verify the correct PFM contraction in real time is needed.

Ultrasonography (US) used as a non-invasive biofeedback method for PFMT. US for PFMT biofeedback is available for the transperineal [12] and transabdominal [13] methods. Transabdominal US is less exposure of the patients than transperineal US and can measure bladder base elevation during PFMT to ensure PFM contraction [14]. Bladder base elevation confirmed by US correlates with perineometry, the gold standard measurement method for PFM contraction [15]. Furthermore, the current US device is a handheld device that works with a smartphone and can be fully utilized for personal use at home for PFMT biofeedback.

Recent studies have demonstrated the feasibility of self-performed US [16–18]. For example, self-performed endovaginal telemonitoring was used for reproductive evaluations during infertility treatment [17], and a study during COVID-19 revealed the feasibility of self-performed lung US in adults at home by teaching remotely [18]. Thus, women with UI may also be able to self-perform US.

However, there is a problem in applying this self-performed US to PFMT biofeedback: US images are difficult to interpret, because reading US images requires specialized knowledge. Therefore, assistance functions are needed to confirm the bladder base elevation. Recently, automatic image processing techniques have been used to analyze US images [19] and an automatic bladder area extraction function for US images has already been developed [20]. There is a need for a method that can automatically assess bladder base elevation due to PFM contraction. A recent study reported automatic evaluation of the bladder during PFM contraction using transperineal US [21], however, transperineal US is not suitable for self-performed US because it is difficult to perform.

Therefore, this study aimed to develop an automatic algorithm for PFM contraction using transverse bladder images obtained by self-performed US to test whether it is applicable to automatically identify PFM contraction from US videos.

## Methods

### Study design and settings

This study conducted a developmental study used US videos collected through a cross-sectional study. We recruited participants from the outpatient Urology and Gynecology departments at a general hospital in Tokyo or by snowball sampling. The study was conducted in multipurpose examination rooms in a hospital or laboratory at a university in Tokyo. Outpatients were collected in a multipurpose room at the hospital, and snowball sampling participants were collected in a laboratory at a university in Tokyo, while maintaining their privacy. One to two researchers accompanied them to collect the data. The study period was from March to November 2022.

 This study was approved by the Research Ethics Committee of the Graduate School of Medicine, University of Tokyo (No. 2021256NI-[2]). Each participant read and gave written informed consent.

### Participants

Women with and without UI were recruited to develop a widely usable algorithm for women. Women aged ≥ 20 years without UI were recruited through snowball sampling, and women aged ≥ 40 years with UI were recruited at outpatient Urology and Gynecology departments at a general hospital in Tokyo, or by snowball sampling. The Japanese version of the ICIQ-SF (International Consultation on Incontinence Questionnaire Short Form) [22], an international questionnaire, was used to confirm UI. Participants who answered “1” or more to Question 1 (How often do you leak urine? ) were considered to have UI. On the other hand, participants who answered “0” were considered to have no UI in this study. We excluded the following women: (1) those with low activities of daily living who were unable to apply US, such as lower activities of daily living as contractures or paralysis in their arms or fingers; (2) those diagnosed with dementia or indicated by a physician; (3) those with skin disease or tenderness in the lower abdomen; (4) women who had undergone cystectomy; (5) pregnant women; (6) women within four weeks of delivery; and (7) physician’s considered inappropriate for participation in the study.

At the hospital, study participants obtained permission from the physician for recruitment. After obtained the physician’s permission, recruitment and consent were obtained from the patients on their outpatient visit.

### US data acquisition and the study protocol

The researcher supported the participants in self-performed bladder US. A handheld US device (iViz air; FUJIFILM, Tokyo, Japan) was used. A 3–5 MHz convex probe was used for bladder imaging at a depth of 15.0 cm in B mode. The self-performed bladder US was performed in the sitting position. The reason for this was that the sitting position was an easy position for performing PFMT and checking US videos.

The participants self-performed the US at least 30 min after the last urination. After showing the image of the bladder on display by self-performed US, the US video recording was started. In response to the researcher’s call, participants performed PFMT 4–10 times. PFM contraction and rest call times were recorded. Approximately one–three recordings were obtained according to the participants’ physical conditions and schedules. When possible, multiple videos were recorded for situations with different urine volumes. Other variables obtained were age, body mass index (BMI), number of deliveries, ICIQ-SF score, overactive bladder symptom score (OABSS) [23], experience with PFMT, and medical history.

### Data processing

The videos obtained from the participants were trimmed based on the contraction time. The trimmed videos were blinded and classified by two experts with sufficient US experience. In this study, these classifications were used as the ground truth data. The classifications were undetermined, correct PFM contraction, PFM contraction failure, and no contraction. If the classifications of the two experts differed, the videos were excluded and considered unsuitable for the analysis.

**Fig. 1:**
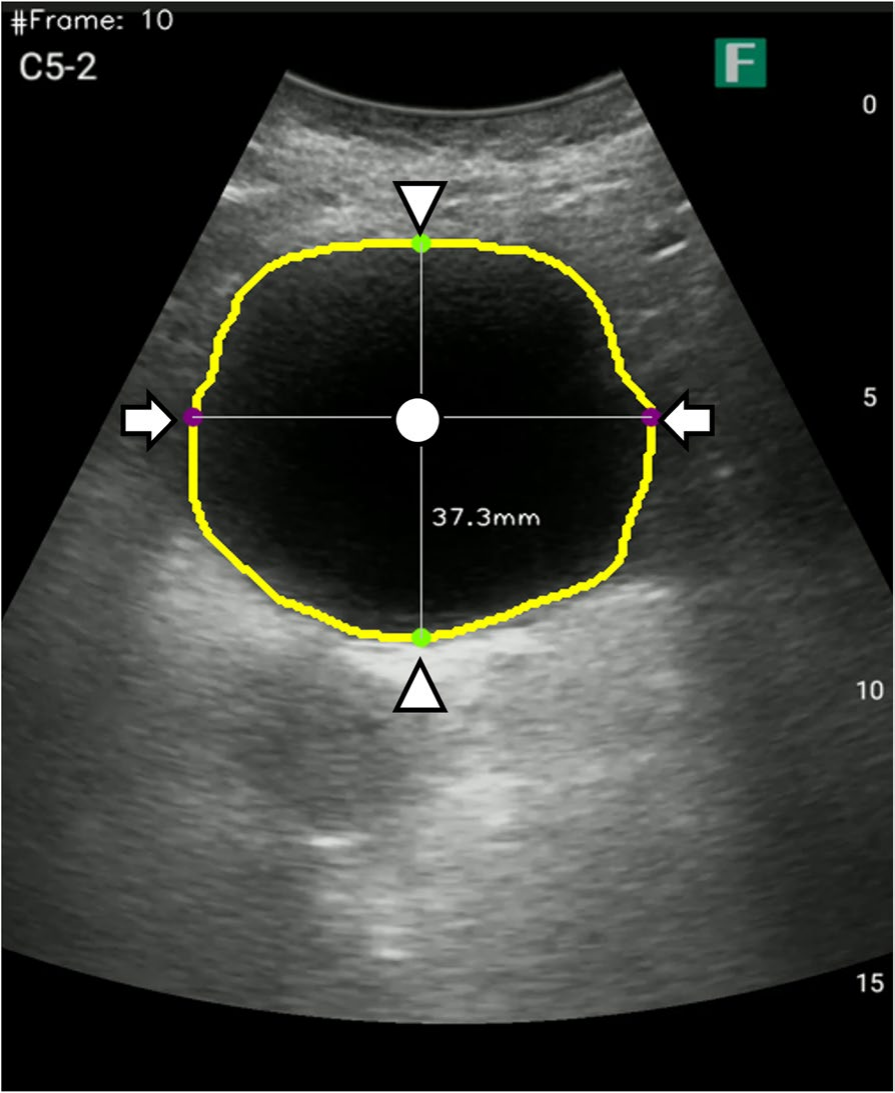
Extracted bladder area. The yellow line surrounding the bladder was the result of bladder area extraction. The white circle point is the center point (Center) of the bladder’s maximum length diameter. Arrow points are the cross point of the bladder wall between the left and right bladder wall and the maximum length diameter (Left, Right). Arrowhead points are the point where the top and bottom bladder walls cross vertically from the center point (Top, Bottom). This US image shows the bladder area was extracted correctly

Specific coordinates were obtained to extract features from the US video of the bladder. The bladder area was extracted from the images using an auto image processing system [20], as shown in Fig. 1. The values of the x- and y-coordinates at five points in the bladder area (center, the center point of the maximum bladder diameter; right/left, the point where the maximum bladder diameter intersects the bladder wall on both sides; top/bottom, the top and bottom of the point where the bladder wall intersects vertically from the center) were automatically extracted. These variables were extracted as time-series data every 10 frames. The videos were recorded at 24 fps.

To generate the time series bladder features, the extracted features from trimmed videos were analyzed with the feature extraction algorithm Tsfresh [24] using Python 3.8.15 by Jupyter Lab. In the Tsfresh, time-series features such as the number of peaks, mean, minimum value, maximum value, and frequency were extracted from the bladder Center, Top, Bottom, Right, and Left. For the other features, the bladder area was calculated from the product of the distance between Right_x and Left_x and the distance between Top_y and Bottom_y as surrogate values for urine volume. The difference between the minimum and maximum values of Bottom_y was obtained as the bladder base elevation.

### Machine learning

The virtual environments used were Pycaret 2.3.1, Numpy version 1.20.3, Pandas version 1.5.2, matplotlib version 3.6.2, scikit-learn version 0.23.2, and Python 3.8.15 by Jupyter Lab. This study used the Pycaret library [25] for supervised machine learning (ML) multiclass classification. An expert PFM contraction classification was used as the ground truth data. Expert classifications were merged with features as labeled data. We then divided the original data into 70% training data and 30% test data. We used five-fold cross-validation to avoid overfitting due to the lack of large datasets [26–28]. After the cross-validation test, 15 classifiers were created and the top two classifiers with the highest accuracy were selected. And Logistic regression was selected as the classical classifier for a reference. Hyperparameter tuning via a grid search approach was performed to optimize the performance of the model, and the classification model with the highest accuracy was selected. The outcome of this study was the performance of the developed algorithm based on the participants’ self-performed bladder videos. The evaluation parameters for this purpose were accuracy, area under the curve (AUC), Recall, Precision, and F1 value. To estimate the contribution of each feature variable to the model’s classification ability, the top 10 feature importance levels were also calculated.

### Statistical analysis

Descriptive statistics were calculated for the participant characteristics. Categorical data were presented as percentages and continuous data were presented as means and standard deviations (SD).

## Results

### US data acquisition

**Fig. 2:**
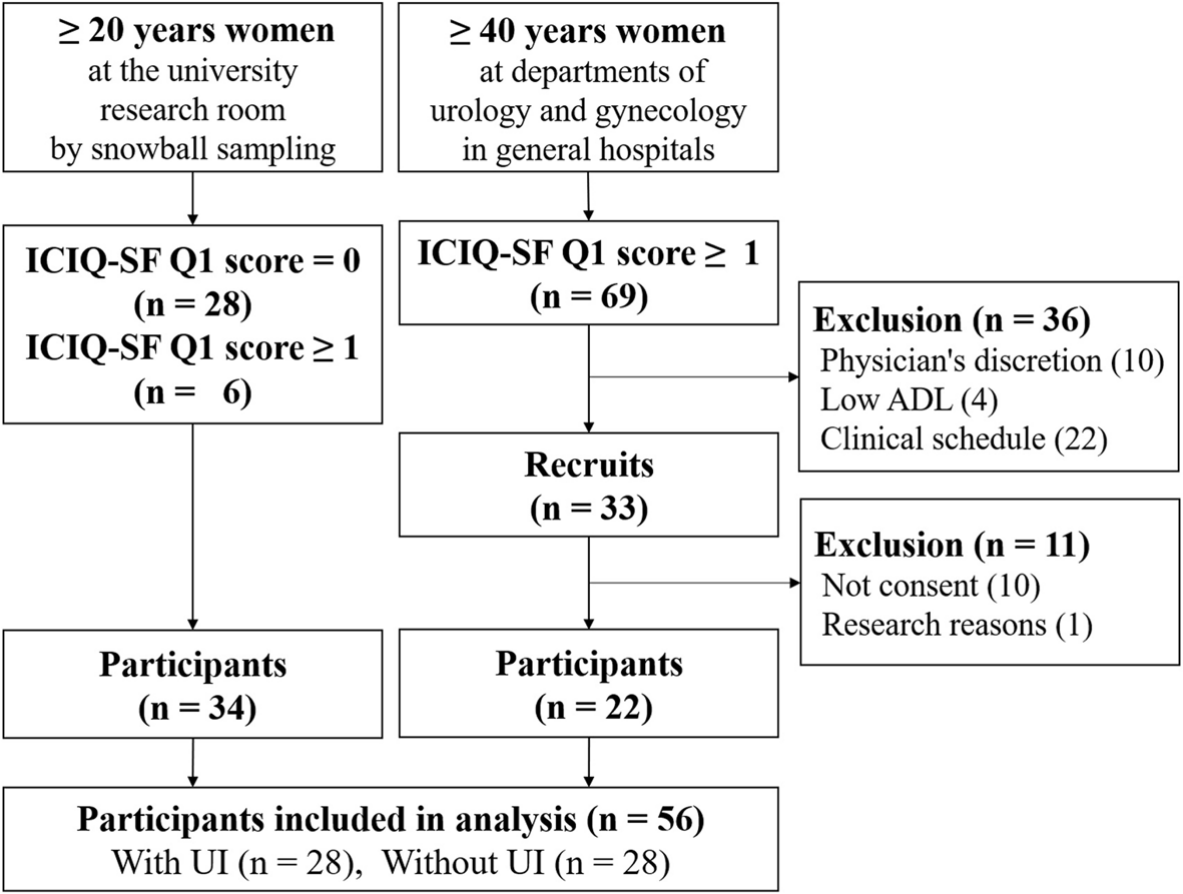
Flow diagram of participants. ICIQ-SF = International Questionnaire Urinary Incontinence Short Form. UI = Urinary Incontinence. ADL = Activities of Daily Living

**Fig. 3:**
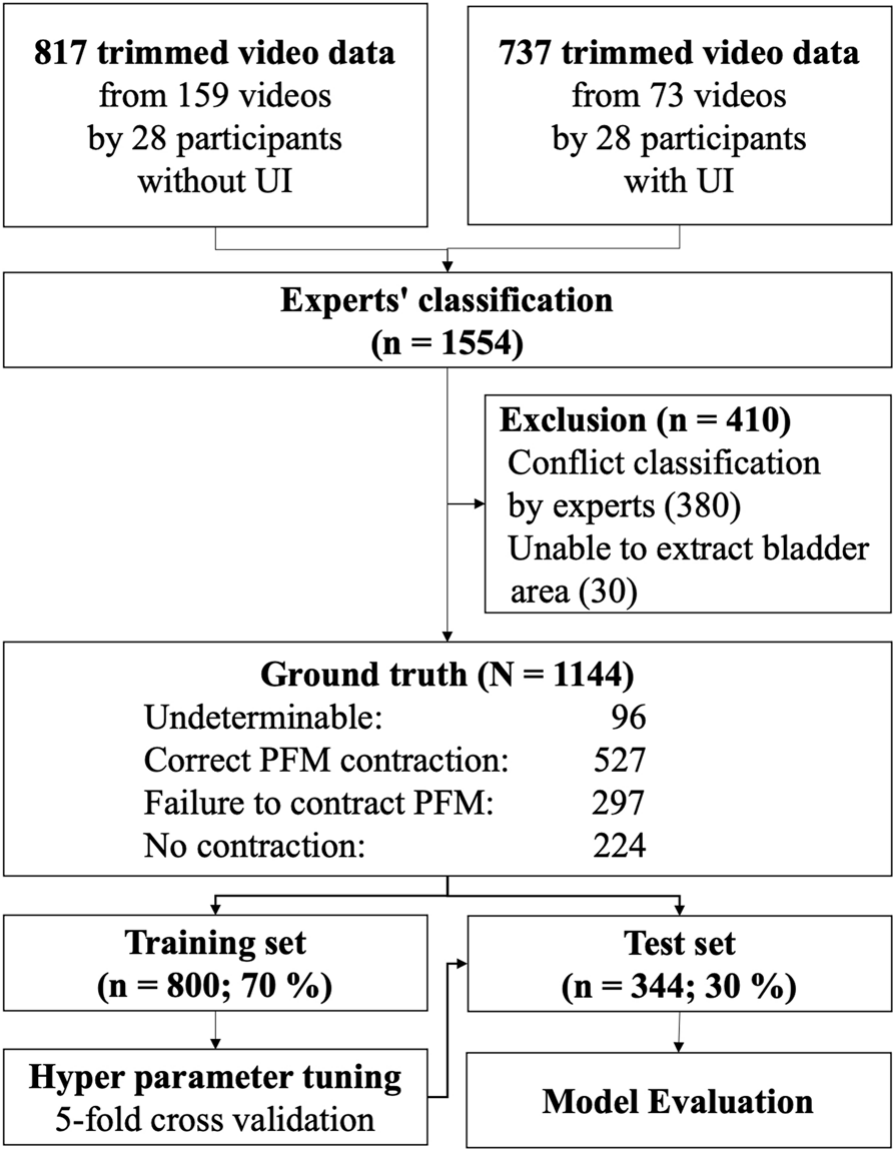
Flow diagram of data for the machine learning process. PFM; Pelvic Floor Muscles. UI; Urinary Incontinence

Fifty-six participants were recruited for the study (Fig. 2). Table 1 presents the characteristics of the participants. A total of 233 bladder self-performed videos were recorded and trimmed to 1554 videos based on the contraction time, excluding videos that were classified differently by experts and researchers. Finally, 1144 videos were analyzed. The experts’ classification was as follows: undeterminable, 96; correct PFM contraction, 527; failure to correct PFM, 297; and no contraction, 224 (Fig. 3).

**Table 1:** Characteristics of participants

	Participants without UI	Participants with UI
	(*n* = 28)	(*n* = 28)
Age (year)	36.0 ± 11.1	62.1 ± 14.5
BMI (kg/m²)	20.2 ± 02.9	22.7 ± 03.5
Delivery (times)	0.4 ± 00.9	1.8 ± 01.2
ICIQ-SF total score	0.0	7.6 ± 03.7
Q1	0	2.0 ± 01.1
Q2	0	2.1 ± 00.7
Q3	0	3.5 ± 02.6
OABSS total score	0.2	5.0 ± 04.3
Q1	0.3 ± 0.5	0.8 ± 00.7
Q2	0.1 ± 0.3	1.3 ± 01.2
Q3	0	1.7 ± 02.4
Q4	0	1.2 ± 01.5
PFMT experience	8 (28.6)	9 (32.1)
History of present illness		
OAB	0 (0.0)	4 (14.3)
UI	0 (0.0)	2 (07.1)
Medical history		
Bladder cancer	0	1 (03.6)
Uterine cancer	0	8 (28.6)
Ovarian cancer	0	1 (03.6)
Hysteromyoma	1 (3.6)	2 (07.4)
Pan hysterectomy	1 (3.6)	7 (25.0)

Data are presented as n (%) or mean ± SD

*Abbreviations: BMI *Body Mass Index (kg/m²), *ICIQ-SF *International Questionnaire Urinary Incontinence Short Form, *OABSS *Overactive Bladder Symptom Score, *PFMT *Pelvic Floor Muscle Training, *OAB *Overactive Bladder Symptom, *UI* Urinary Incontinence, *SD* Standard Division

### Extraction features and machine learning

**Fig. 4:**
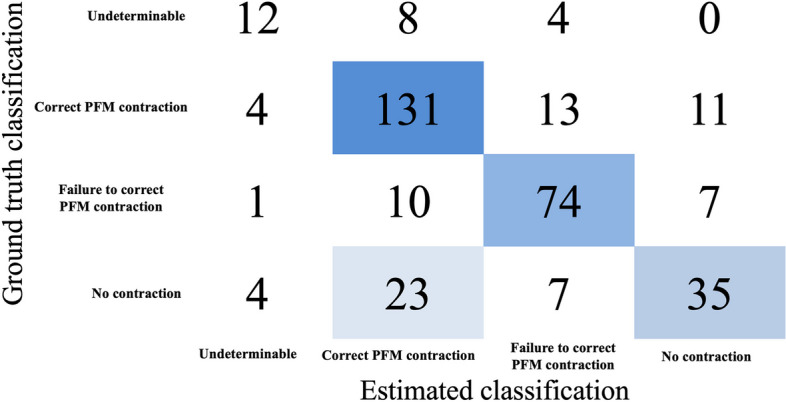
Confusion matrix for each estimation by LightGBM model in test data set. PFM; Pelvic Floor Muscles. LightGBM; Light Gradient Boosting Machine

From 1144 videos for the training dataset (70%) and test dataset (30%). A total of 10,257 features were extracted. ML was performed with 7,894 features after excluding those with missing values (Fig. 3). As a result of classifier comparisons after the five-fold cross-validation test, the light gradient boosting machine (LightGBM) [28] and the random forest classifier showed high accuracy. The logistic regression accuracy was 0.47 (Table 2). LightGBM was selected because the highest accuracy and the other indices were also good, and the hyperparameters were tuned using a grid search in a five-fold cross-validation. The final classification model results were as follows: accuracy = 0.73, AUC = 0.91, Recall = 0.66, Precision = 0.73, and F1 = 0.73. The results for each classification are shown in Fig. 4 as a confusion matrix. In feature importance, the fast Fourier transformed Bottom_y feature produced the highest results. In addition, 5 of the top 10 features were related to Bottom_y. The top three features indicated the y-coordinates of the upper and lower portions of the bladder.

**Table 2:** Performance of each model based on 5-fold cross-validation

Model	Accuracy	AUC	Recall	Precision	F1
Light Gradient Boosting Machine	0.73	0.89	0.66	0.72	0.72
Random Forest Classifier	0.73	0.88	0.64	0.73	0.71
Logistic Regression	0.47	0.61	0.30	0.35	0.39

Accuracy: (TP + TN)/(TP + FP + FN + TN), Recall: TP/(TP + FN), Precision: TP/(TP + FP), F1: 2 * (Precision * Recall)/Precision + Recall

*AUC* The Area Under the Curve, *TP *True Positive, *TN *True Negative, *FP *False Positive, *FN* False Negative

## Discussion

In this study, an estimate was developed to automatically determine the feedback of PFMT using ultrasound, a procedure that previously required advanced reading skills and could only be performed in hospitals by experts. The model was developed through supervised ML using the expert opinion of a sonographer as the ground truth, thus achieving a high level of performance with an accuracy of 0.73 and an AUC of 0.91. These results indicate the possibility of automatic evaluation of PFM contraction from US videos, similar to previous studies [21]. The novelty of this study is that it used transabdominal, transverse bladder videos, compared to previous study that used transperineal US videos [21]; automatic evaluation of contraction of PFM in four categories.; and the US movies used were all obtained from the participant’s self-performed.

In this study, supervised ML was applied to 1144 US videos of adult women. This model enabled four classifications (undeterminable, correct PFM contraction, failure to contract PFM, and no contraction) with a high accuracy. This model may help determine not only whether the PFMT was successful, but also in what way the contraction went wrong to help the patient understand how to modify his or her PFMT. For the 23 video images, the model classification exhibited correct PFM, although they were not properly contracted. These videos showed no evidence of pelvic floor muscle contraction; however, they contained abdominal wall movement due to abdominal breathing and subtle changes in probe movement, which may have led to misclassification due to changes in bladder position. Further improvement of the estimation performance is expected in the future by improving the system to be able to distinguish between fine movements such as respiratory variation, as well as by incorporating compensation for camera shake on the probe side.

Five of the top ten characteristics identified in this study were related to the bladder base. In previous studies, expert feedback using US images suggested the importance of bladder base motion, this result is consistent with the clinical findings and feature importance analysis [29, 30]. The most important feature was associated with the fast Fourier transform, which is a numerical representation of the amplitude and position of the waveform based on frequency component decomposition, representing the complexity of the time-series data. The top three features indicated the y-coordinates of the upper and lower portions of the bladder, and variations in the upper and lower portions of the bladder were significant determinants of PFM contraction.

This study has a few limitations. First, the number of participants who were diagnosed with UI or overactive bladder was small. Therefore, the ML dataset included only a limited number of bladder US videos obtained during PFMT for patients diagnosed with UI or overactive bladder. In order to utilize this algorithm as biofeedback for initial training for patients with severe symptoms, more US videos of patients with UI and overactive bladder should also be learned in the future. Second, the number of US videos obtained in this study was small. This was due to the limited recruitment period. However, conducting further analysis with an expanded dataset could potentially enhance the predictive accuracy of PFM contraction in future studies. In addition to the participants characteristics obtained in this study, additional personal data such as delivery information, smoking, and alcohol consumption, which could be attributed to urinary incontinence, could be obtained to develop an automated pelvic floor muscle evaluation system that is more clinically relevant. In addition, more than half of the participants in this study were recruited through snowball sampling. This was conducted because PFMT is not only used to treat UI symptoms but also as a preventive measure, and therefore we aimed to develop a more general-purpose system by recruiting community women as well as hospitals. Although snowball sampling may have introduced a selection bias in the target population, this study ultimately combined data obtained by snowball sampling and data obtained at the hospital to perform ML. Therefore, we believe that we developed an algorithm that excludes bias as much as possible.

This study revealed that four classifications of PFM contractions are possible based on women’s self-performed US videos. In this study, we used ML with bladder coordinate data for automatic evaluation of PFM contraction. This was because we thought that this would be the variable that would provide the best understanding of bladder movement in this new attempt to estimate movement from bladder videos acquired by self-performed US. However, there are other approaches that can estimate PFM contraction from bladder US videos, such as Convolutional Neural Network (CNN) and Vision Transformer. Although these methods require large amounts of data, they were not implemented in this study because the amount of data was limited. Additional research should be conducted in the future by adding more data and examining analysis techniques to further improve the accuracy of PFMT classification. Furthermore, the feasibility and effectiveness of this system for UI symptoms needs to be established.

The biofeedback method proposed in this study, combined with self-performed US, suggests that women with UI can receive PFMT biofeedback at home at any time. This is expected to help women maintain their health by improving and preventing UI and ultimately extending their healthy life expectancy through UI independence.

## Conclusion

In this study, supervised ML was performed using bladder videos obtained from women’s self-performed US in order to develop an algorithm for automatic evaluation of PFM contractions. The results showed that the algorithm could automatically classify PFM contractions with a high performance of 0.73 accuracy and 0.91 AUC. The results of this study suggest a new biofeedback method for women requiring PFMT.

## Data Availability

The datasets generated and/or analyzed during the current study are not publicly available due to the lack of consent from participants for the public release of research data, as well as the ongoing nature of the study which involves sensitive and confidential information but are available from the corresponding author on reasonable request.
